# Sintilimab: A Promising Anti-Tumor PD-1 Antibody

**DOI:** 10.3389/fonc.2020.594558

**Published:** 2020-11-26

**Authors:** Lin Zhang, Wuqian Mai, Wenyang Jiang, Qing Geng

**Affiliations:** ^1^ Department of Thoracic Surgery, Renmin Hospital of Wuhan University, Wuhan, China; ^2^ Department of Cardiology, Union Hospital, Tongji Medical College, Huazhong University of Science and Technology, Wuhan, China; ^3^ Key Lab of Molecular Biological Targeted Therapies of the Ministry of Education, Union Hospital, Tongji Medical College, Huazhong University of Science and Technology, Wuhan, China

**Keywords:** sintilimab, programmed cell death protein 1(/programmed cell-death protein 1 ligand 1, immunotherapy, non-small cell lung cancer, solid tumors, Hodgkin lymphoma

## Abstract

Sintilimab (Tyvyt^®^) is a monoclonal antibody against programmed cell death protein 1 (PD-1). It could block the interaction between PD-1 and its ligands and help the anti-tumor effect of T-cells to recover. Sintilimab is developed by Innovent Biologics and Eli Lilly and Company and has been approved to treat relapsed or refractory classical Hodgkin lymphoma in patients who have undergone two or more lines of systemic chemotherapy by the National Medical Products Administration of China. Recently, sintilimab has been reported in plenty of literature and shows satisfying anti-tumor effect. Meanwhile, there are some reports showing its side effects. Overall, sintilimab has similar anti-tumor effects and a better safety profile compared to nivolumab and pembrolizumab in Hodgkin lymphoma, natural killer/T cell lymphoma and advanced non-small cell lung cancer. In this review, we aim to briefly describe the mechanisms, pharmacological characteristics, anti-tumor effects, predictive parameters of efficacy and side effects of sintilimab, providing valuable information of sintilimab for decision-making in the treatment of tumors in the future.

## Introduction

Immune checkpoints (ICs) are important immune regulators in maintaining immune homeostasis and preventing autoimmune diseases. There are co-stimulatory ICs, like CD28 and TNFRSF9, and inhibitory ICs, like CTLA-4 (1). Under normal conditions, ICs allow the immune system to respond to infections, malignancies and protect tissues. However, the expression of some immune checkpoint proteins by tumor cells disturbs the anti-tumor immunity of the body, facilitating the growth and expansion of malignant cells (2). Thus, blockade of the immune checkpoint pathway using immune checkpoint inhibitors (ICIs) is a weapon in cancer therapy. Among all ICIs, the most studied ones are anti-programmed cell death protein 1(PD-1)/programmed cell-death protein 1 ligand 1(PD-L1) antibodies (3).

Sintilimab is a fully human IgG4 monoclonal antibody that is registered as Tyvyt. It was developed by Innovent Biologics and Eli Lilly and Company (4). Sintilimab is able to bind to PD-1, block the interaction of PD-1 with its ligands, and help to recover the anti-tumor response of T-cells. By 2020, sintilimab has been approved by the National Medical Products Administration (NMPA) of China to treat relapsed or refractory classical Hodgkin lymphoma (r/r cHL) (5). Compared with nivolumab and pembrolizumab, two well-studied PD-1 inhibitors approved by China NMPA and the US Food and Drug Administration (FDA), sintilimab has a similar anti-tumor effect, a better safety profile, and obvious economic advantages. Here, we summarized the mechanisms, pharmacological characteristics, anti-tumor effects, predictive parameters of efficacy and adverse effects (AEs) of this drug to provide a potential valuable choice for decision-making in the treatment of tumors in the future.

## PD-1/PD-L1 Pathway

PD-1 was first characterized in 1992 by Ishida et al. of Kyoto University in Japan (6). As one of the immune checkpoint membrane proteins, PD-1 is synthesized in the endoplasmic reticulum and then delivered to the cell membrane surface to play inhibitory roles (3). Structurally, PD-1 is a type I transmembrane protein that belongs to the CD28 immunoglobulin (Ig) subfamily and is an important immunosuppressive protein (7). PD-1 consists of an Ig-V like extracellular domain, a transmembrane domain, and a cytoplasmic domain; the cytoplasmic domain is comprised of two tyrosine signaling motifs, which are known as immunoreceptor tyrosine inhibitory motif (ITIM) and immunoreceptor tyrosine-based switch motif (ITSM) (6, 8). Previous research has indicated that the tyrosine within the ITSM motif is indispensable for the function of PD-1 in T cells and B cells (9, 10). PD-1 could recruit protein tyrosine phosphatase SHP2/SHP1 after binding with its ligands; the process was mediated *via* phosphorylated ITSM/ITIM, which could in turn inhibit both T cell receptor (TCR) and CD28 (3, 11). The downstream phosphatidylinositol 3 kinase (PI3K)/protein kinase B (AKT) pathway of CD28 and signaling molecules like zeta-chain associated protein 70 of TCR were then inhibited (3). The ligands of PD-1 included PD-L1 and PD-L2 (12, 13). The expression of PD-L2 on tumor cells is rare, and the role of PD-1/PD-L1 pathway in the regulation of anti-tumor immunity is more studied (14).

The PD-1/PD-L1 pathway is involved in the regulation of tumor progression in many aspects. The expression of PD-L1 was mainly induced by interferon-*γ* (IFN-*γ*), and the primary mechanism of PD-1/PD-L1 pathway blockade is generally thought to be the priming of tumor-specific cytotoxic T cells in the tumor microenvironment (11, 15). The binding of PD-L1 and PD-1 could lead to T cell apoptosis, anergy, exhaustion, and interleukin-10 expressions (15, 16). PD-L1 may also function as a molecular “shield” to protect malignant cells from cytotoxic responses mediated by CD8^+^ T cells (15, 17). Activated T cells and antigen-presenting cells could express CD80, a receptor of PD-L1, delivering inhibitory signals when binding to it (16). In addition, PD-L1 might act as a receptor to transmit signals from T cells to tumor cells, resulting in their resistance to lysis (17). Tumor-infiltrating lymphocyte (TIL) is a major effector cell in the microenvironment of tumor tissues, and it highly expresses PD-1 molecules on the surface (15). TIL inactivation is a consequence of PD-1/PD-L1 activation since silencing PD-1 and PD-L1 could effectively increase TIL cytotoxicity to cancer cells (18). Finally, the overexpression of PD-1 in tumor cells could promote the growth of tumors (19).

## Pharmacological Characteristics of Sintilimab

Sintilimab has an effective anti-tumor activity. *In vitro*, the binding affinity of sintilimab was high with a low dissociation constant (KD) (20, 21). Correspondingly, sintilimab has shown a higher level of PD-1 occupancy than nivolumab and pembrolizumab in peripheral blood mononuclear cells (PBMCs) and mice reconstituted with PBMCs (20). In patients, a sustained PD-1 receptor occupancy of more than 95% for up to 4 weeks was observed after a single sintilimab infusion intravenously (20). Sintilimab could effectively activate T cells, as evidenced by the enhanced interleukin-2 and IFN-*γ* secretion among T cells co-incubated with it (20, 21). PD-1 is inducibly expressed on activated T cells but is not expressed on resting T cells (22). In a human tumor xenograft mice model reconstituted with human immune cells or a PD-1 knockin tumor mouse model, sintilimab could significantly inhibit tumor growth and the effect was more prominent than equal doses of nivolumab or pembrolizumab (20, 21).

Sintilimab could be metabolized effectively and has an acceptable safety profile in the body. The serum half-life (t_1/2_) of sintilimab is 35.6 h compared to those of 43.5 h and 42.5 h for nivolumab and pembrolizumab, respectively (20). The concentration for 50% of maximal effect (EC50) is 2.2 nM (21). Like other therapeutic antibodies, it has an IgG4 backbone, which is known to have a very low effector function. In fact, sintilimab has no or weak affinity to Fc receptors for IgG (Fc*γ*R), implicating that sintilimab does not induce antibody or complement-dependent cytotoxic responses (21). Also, sintilimab has a weak immunogenicity as reflected by low detection rates of anti-drug antibodies (0.52%, 2/381) and neutralizing antibodies (0.26%, 1/381) in patients. The toxicity of sintilimab was tolerable in a 26-week toxicity study in which sintilimab was administrated biweekly up to 200 mg/kg and caused no death among cynomolgus monkeys (21). However, a potential risk of IgG4 antibodies is Fab-arm exchange (FAE), which means the swap of a heavy chain and the attachment of light chain with a heavy-light chain pair from another molecule in IgG4 antibody; FAE could lead to molecular instability (23). Luckily, FAE could be eliminated by antibody engineering with a single point mutation (24).

## Efficacy of Sintilimab in Tumors

In a first-in-human phase I trial (NCT02937116), sintilimab has shown preliminary efficacy and tolerance among 12 patients with solid tumors including hepatocellular carcinoma and neuroendocrine tumor (25). In that study, sintilimab was given at escalating doses: 1, 3, 200 (1:1 randomization) and 10 mg/kg. Finally, a dose of 200 mg per 3 weeks (q3w) was recommended for further studies (25).

### Efficacy of Sintilimab in Lymphoma

Sintilimab monotherapy has shown anti-tumor efficacy in lymphoma. The first indication of sintilimab is relapsed or refractory classical Hodgkin lymphoma after a second-line chemotherapy, which was a conditioned approval based on a multicenter, single-arm, phase II trial (ORIENT-1) (26, 27). In ORIENT-1, 96 patients with r/r cHL after ≥2 lines of therapy from 18 hospitals in China were incorporated with 92 included in the full analysis (26). Patients were given intravenous sintilimab 200 mg q3w until progression, death, unacceptable toxicity, or withdrawal of consent; among them, 74 patients (80.4%; 74/92) had an objective response (OR), defined as complete or partial remission on CT or MRI, according to an independent radiological review committee (26, 28). In comparison, 66.3% (53/80) and 69.0% (145/210) r/r cHL patients achieved OR after treatment with nivolumab (3 mg/kg every 2 weeks) and pembrolizumab (200 mg, q3w), respectively; these patients were treated until disease progression, intolerable toxicity, or withdrawal by investigator decision, as reported by phase II studies CHECKMATE-205 and KEYNOTE-087, correspondingly (29, 30). In another phase II trial ORIENT-4, sintilimab monotherapy showed an anti-tumor effect in extranodal NK/T cell lymphoma (ENKTL) (31). In 28 enrolled patients, sintilimab was given at 200 mg intravenously q3w until disease progression, death, unacceptable toxicity, or withdrawal from the study; the OR rate (ORR) was 67.9% (19/28); disease control rate (DCR) was 85.7% (24/28) (31). In contrast, pembrolizumab achieved a total ORR of 78.6% (11/14) among 14 ENKTL patients in two retrospective studies (32, 33). Although the efficacy of both drugs seems similar, the conclusion about their efficacy on ENKTL may not be rigorous because of the small sample sizes. In addition, sintilimab was also reported to be used in a human immunodeficiency virus-infected r/r cHL patient, with a satisfying efficacy and mild, acceptable toxicities (34). In diffuse large B-cell lymphoma, a triple combination of decitabine, sintilimab plus a histone deacetylase inhibitor chidamide achieved partial remission (PR) (35).

### Efficacy of Sintilimab in Non-Small Cell Lung Cancer

In non-small cell lung cancer (NSCLC), sintilimab monotherapy has also proved effective. In a phase Ib study (ChiCTR-OIC-17013726), 40 patients with resectable NSCLC were enrolled and given two doses of intravenous sintilimab (200 mg) q3w, followed by an operation; neoadjuvant sintilimab resulted in an ORR of 20.0% (8/40) and a DCR of 90% (36/40) (36). Among 37 patients undergoing radical resection, 15 (40.5%) achieved major pathological response (MPR), defined as tumors with 10% viable tumor cells or less; the 37 patients included six (16.2%) with a pathologic complete response (pCR) in primary tumor and three (8.1%) in lymph nodes as well (36–38). In NCT02259621, two preoperative doses of nivolumab (3 mg/kg) were administered every 2 weeks among 21 patients with stage I, II, or IIIA NSCLC; neoadjuvant nivolumab resulted in an ORR of 9.5% (2/21) and a DCR of 95.2% (20/21) among enrolled patients, with 42.9% (9/21) achieving MPR (39). In a phase I study MK3475-223, two doses of pembrolizumab (200 mg) was administered in stage I and II NSCLC patients, achieving an MPR of 40% (4/10) (40, 41). Sintilimab seems to have a similar efficacy to nivolumab and pembrolizumab in resectable NSCLC patients when used preoperatively. However, the conclusion may need further evidence in consideration of the small sample sizes.

In addition, sintilimab plus chemotherapy has a strong anti-tumor activity in NSCLC. A multicenter, phase Ib study involving 20 patients in China (NCT02937116) has shown the safety and well tolerance of sintilimab with a satisfying efficacy (42). In this study, sintilimab 200 mg q3w in combination with gemcitabine and cisplatin regimen was administered in treating naive patients with advanced squamous cell NSCLC until disease progression or intolerant toxicity, yielding a 64.7% (11/17) ORR, a 30% (6/17) stable disease (SD) proportion among those who have received at least one radiological assessment (42). Most recently, a randomized, double-blind, phase III study (ORIENT-11) involving 397 stage IIIB to IV non-squamous NSCLC patients with no previous systemic treatment has shown that the combination of sintilimab and pemetrexed plus platinum has led to significantly longer progression-free survival (PFS) than that of chemotherapy alone with manageable adverse effects (43). In ORIENT-11, patients received either sintilimab 200 mg or placebo plus pemetrexed and platinum q3w for 4 cycles as induction therapy, followed by sintilimab or placebo plus pemetrexed as maintenance therapy q3w for up to 24 months; although median overall survival (OS) was not reached, the median PFS, ORR, DCR were 8.9 months, 51.9% (138/266), 86.8% (231/266) in the sintilimab-combination group compared to those of 5.0 months, 29.8% (39/131) and 75.6% (99/131) in the placebo-combination group (placebo plus chemotherapy) (43). In contrast, in a phase III study KEYNOTE-189, previously untreated patients with metastatic non-squamous NSCLC were also assigned to receive pemetrexed and platinum plus pembrolizumab (n = 410) or placebo (n = 206) q3w for 4 cycles; the median PFS, median OS, ORR, and DCR of patients in the pembrolizumab group were 9.0 and 22.0 months, 48.0% (197/410), 84.6% (347/410), compared to those of 4.9 and 10.7 months, 19.4% (40/266), 70.4% (145/206) of patients in the placebo group (44). By comparison, sintilimab plus chemotherapy has shown similar PFS, ORR, DCR to pembrolizumab and was demonstrated effective.

In single cases, sintilimab was also reported effective in NSCLC. The combination of sintilimab and bevacizumab has led to shrinkage in tumor of a 53-year-old male patient with metastatic adenocarcinoma and resulted in PFS of 6.0 months (45). Also, in ChiCTR-OIC-17013726, one patient with a radiographic assessment of progressive disease (PD) according to RECIST 1.1 was identified with a 60% pathologic remission at primary tumor site after operation; two patients with radiographic response achieved 100% pathologic remission at the primary tumor site (36, 46). The sign of the PD patient in ChiCTR-OIC-17013726 was named pseudoprogression, indicating that the patient who was evaluated as PD according to RECIST 1.1 was actually undergoing late but durable responses (47). In our previous report, pseudoprogression and 100% pathologic remission were presented in an NSCLC case (48). In that report, a 64-year-old woman diagnosed with initially unresectable squamous NSCLC was given sintilimab plus nedaplatin and paclitaxel for three cycles before surgery; the patient achieved pCR and remained disease-free in the following several-month follow-up (48). In another report, a 65-year-old female patient with unresectable hepatoid adenocarcinoma, docetaxel plus sintilimab has resulted in an SD and a significant PR later on (49). Moreover, sintilimab was reported to maintain effective in a patient more than 3 months after drug withdrawal (50). Because of the satisfying efficacy of sintilimab on NSCLC, the National Medical Products Administration of China has officially accepted the application for the use of sintilimab plus gemcitabine as the first-line therapy in advanced NSCLC (51, 52). There may likely be an approval for the use of sintilimab in treating NSCLC in the near future.

### Efficacy of Sintilimab in Digestive System Cancers

In digestive system cancers, sintilimab showed favorable anti-tumor effects as well. In a phase I study NCT02937116, 20 gastric/gastroesophageal junction adenocarcinoma (G/GEJ) patients were enrolled; among them, 200 mg sintilimab was administered intravenously in combination with CapeOx (1,000 mg/m^2^ capecitabine orally, bid, days 1–14 and 130 mg/m^2^ oxaliplatin intravenously, day 1) every 21 days for up to six cycles; after combination treatment, patients continued to receive sintilimab (200 mg) at 3 weekly intervals as maintenance therapy until progressive disease, unacceptable toxicity, withdrawal of informed consent, or for up to 24 months (53). The combination therapy resulted in PFS of 7.5 months, an ORR of 85% and a DCR of 100% (53). In comparison, nivolumab in combination with CapeOx until disease progression, unacceptable toxicity, or consent withdrawal resulted in PFS, ORR, DCR of 10.6 months, 76.5% (13/17) and 88.2% (15/17) in such patients (54). The combination of CapeOx and sintilimab seems to be superior to that of CapeOx and nivolumab in terms of ORR and DCR although it has shorter PFS. However, the conclusion needs further evidence since the sample sizes are small.

In a phase II study ORIENT-2, 190 advanced esophageal squamous cell carcinoma (ESCC) patients refractory to first-line chemotherapy were randomly assigned (1:1) to receive sintilimab (200 mg, q3w) or chemotherapy (paclitaxel, 175 mg/m2, q3w; or irinotecan, 180 mg/m2, per 2 weeks), intravenously (55). The median OS was 7.2 *versus* 6.2 months, and the ORRs were 12.6% (12/95) *versus* 6.3% (6/95) (55). In comparison, ORRs of patients who were refractory to first-line chemotherapy and received pembrolizumab (200 mg q3w) or nivolumab (240 mg every two weeks) alone until disease progression, unacceptable toxic effects, or study withdrawal were 14.3% (9/63) and 19.3% (33/171), respectively; their median OS was 6.8 and 10.9 months (56, 57). The ORR of sintilimab is inferior to nivolumab and pembrolizumab, while its median OS is between them. In a phase Ib study NCT04072679, sintilimab was combined with IBI 305, a biosimilar candidate of bevacizumab, to treat hepatocellular carcinoma (HCC); in that study, sintilimab 200 mg plus IBI 305 7.5 mg/kg or IBI 305 15 mg/kg q3w was applied in two groups of advanced or metastatic HCC patients, achieving ORRs of 24.1% (7/29) and 33.3% (4/21), respectively (58). However, when combined with fruquintinib, a vascular endothelial growth factor receptor (VEGFR) inhibitor, sintilimab seemed not to have resulted in a significant increase in ORR, DCR, and OS in refractory metastatic colorectal cancer (59).

In single cases, sintilimab has also shown anti-tumor effect in digestive system cancers. In a 46-year-old male patient diagnosed with HCC, lung metastasis was confirmed after resection of the right anterior section of the liver and intra-abdominal metastasis (60). Sintilimab and IBI305 were applied and lead to complete remission (CR) (60). Besides, in a 54-year-old female patient with metastatic intrahepatic cholangiocarcinoma (ICC), sintilimab was provided after the failure of the first-line treatment of gemcitabine plus cisplatin chemotherapy; CR was achieved after three cycles of treatment (61). In another case, a 63-year-old woman was diagnosed with stage IV ICC involving multiple sites, PR was achieved after six cycles of combined therapy with sintilimab injection and tegafur–gimeracil–oteracil potassium capsules (62). Sintilimab was also reported to exert anti-tumor effects in metastatic gastric cancer and esophageal cancer, leading to the repression of tumor progression and a decrease in serum tumor markers (63). Considering the reported effect in these cases and ongoing studies such as NCT04072679 (58), digestive system cancers like HCC and ESCC might also be indications of sintilimab in the future.

### Efficacy of Sintilimab in Other Tumors

In other tumors, sintilimab was also reported to be effective. In a prospective study analyzing the efficacy and safety of sintilimab in late stage solid tumors, including melanoma, lung cancer, digestive system caners, and neuroendocrine carcinoma, CR and PR were achieved in two (3.8%, 2/53; one melanoma and one NSCLC) and 21 (39.6%, 21/53; one neuroendocrine carcinoma, 10 NSCLC and 10 G/GEJ) patients, respectively (64). In the study, sintilimab was administered at a dose of 200 mg q3w alone or with first-line chemotherapy until disease progression, intolerable toxicity or withdrawal by investigators’ decision (64). The median PFS in this study was 118 (95% CI, 96–140) days, and median OS was not reached. In another retrospective single center study involving 10 patients with advanced renal cell carcinoma, sintilimab plus axitinib leads to a 40.0% ORR (4/10) and a 90.0% DCR (9/10) (65). The characteristics of major clinical studies mentioned above are listed in **Table 1**. In addition, there are ongoing trials of sintilimab on various kinds of cancers, such as those on NSCLC and hepatocellular carcinoma (66). Their results might lead to new indications of this drug in the future.

**Table 1:** Summary of major clinical studies of sintilimab.

Clinical studies	ORIENT-1	ORIENT-2	ORIENT-4	ORIENT-11	ChiCTR-OIC-17013726	NCT02937116	NCT02937116	NCT02937116	NA (Jin Liu)
Cancer type	r/r cHL	ESCC	ENKTL	NSCLC	NSCLC	ST	NSCLC	G/GEJ	ST
Numbers of cases	96	190 (n = 95 per group)	28	397	40	12	20	20	63
Therapeutic regimen	sintilimab	sintilimab *vs*. chemotherapy	sintilimab	sintilimab plus chemotherapy *vs*. chemotherapy	sintilimab (neoadjuvant)	sintilimab	sintilimab plus chemotherapy	sintilimab plus chemotherapy	Sintilimab alone (mono) or plus chemotherapy (comb)
ORR	80.4% (74/92)	12.6 *vs*. 6.3%	68% (19/28)	51.9% (138/266) *vs*. 29.8% (39/131)	20.0% (8/40)	18.2% (2/11)	64.7% (11/17)	85.0% (17/20)	18.5% (5/27, mono), 69.2% (18/26, comb)
DCR	97.8% (90/92)	NA	85.7% (24/28)	86.8% (231/266) *vs*. 75.6% (99/131)	90.0% (36/40)	36.4% (4/11)	100% (17/17)	100% (20/20)	66.7% (18/27, mono), 96.2% (25/26, comb)
TRAEs	54.0% (52/96)	54.3 *vs*. 90.8%	100% (28/28)	99.6% (265/266) *vs*. 100% (131/131)	52.5% (21/40)	NA	100% (20/20)	100% (20/20)	79.3% (50/63)
The most common TRAEs	Pyrexia(38%, 36/96)	NA	NA	Anemia (74.1%, 197/266 *vs*. 78.6%, 103/131)	Asthenia(17.5%, 7/40), hypothyroidism(17.5%, 7/40)	Fever, thyroid dysfunction, serum bilirubin elevation and pneumonitis (25.0%, 3/12 each)	Rash (15.0%, 3/20)	Decreased platelet count(80%, 16/20)	Altered electrocardiogram (32.3%, 10/31, all grade 1-2, mono), hematologic abnormalities (65.6%, 21/32, comb)
Grade 1–2 TRAEs	51.0% (49/96)	34.1 *vs*. 51.7%	60.7% (17/28)	38.0% (101/266) *vs*. 41.2% (54/131)	42.5% (17/40)	NA	30.0% (6/20)	45% (9/20)	65.1% (41/63)
Grade 3–4 TRAEs	3.1% (3/96)	NA	39.3% (11/28)	59.4% (158/266) *vs*. 51.9% (68/131)	7.5% (3/40)	33.3% (4/12)	70.0% (14/20)	55% (11/20)	16.1% (5/31, mono), 12.5% (4/32, comb)
Grade 5 TRAEs	0	NA	0	2.3% (6/266) *vs*. 6.9% (9/131)	2.5% (1/40)	0	0	0	0

In some studies, numbers of assessable patients were less than the numbers of cases.

NA, not available/applicable; r/r cHL, relapsed or refractory classical Hodgkin lymphoma; ESCC, esophageal squamous cell carcinoma; ENKTL, extranodal NK/T cell lymphoma; ST, solid tumors; NSCLC, non-small cell lung cancer; G/GEJ, gastric/gastroesophageal junction adenocarcinoma; vs., versus; ORR, overall survival rate; DCR, disease control rate; TRAE, treatment-related adverse effect; ALT, alanine transaminase; AST, aspartate transaminase.

## Predictive Parameters of Sintilimab Efficacy

In the above-mentioned studies, some indexes were shown to be predictive of sintilimab efficacy. In a biomarker study involving 75 participants from ORIENT-1, circulating tumor DNA (ctDNA) was proved to be a potential biomarker for sintilimab immunotherapy (5). Among patients with a ctDNA, a decrease of ≥40 and 79.31% (23/29) had an agreement between the change in ctDNA and the best radiographic response, with CR and PR achieved in 90% (5). Also, patients whose tumor area decrease is equal or larger than 60% exhibited a more substantial ctDNA decline than those whose tumor area decrease is less than 60% (5). In ChiCTR-OIC-17013726, squamous cell NSCLC exhibited a better pathological response to sintilimab than did adenocarcinoma (MPR: 48.4 *versus* 0%) (36). Moreover, a significant correlation was observed between maximum standardized uptake values (SUVmax) reduction and pathologic response (36). In 2009, traditional SUV was replaced by standard uptake value of lean body mass (SUL) in the PERCIST 1.0 standard which was proposed for evaluating the efficacy of solid tumors with positron emission tomography (PET)-computer tomography (CT) (67). Thus, the correlation between SULmax, a replacement of SUVmax, and the pathological response of 36 participants in ChiCTR-OIC-17013726 was analyzed in another study by Tao et al. (68). The study demonstrated that SULmax of PET-CT performed at baseline was positively correlated with the degree of pathological regression of primary tumor; the study also showed that all metabolic parameters of PET-CT performed within 1 week prior to surgery and the percentage changes of metabolic parameters after neoadjuvant therapy were negatively correlated with the regression of tumor (68).

## Adverse Effects of Sintilimab

Although sintilimab has shown a potent efficacy in the treatment of tumors, its side effects and its potential damage to patients are inevitable. In the official instructions, the adverse effects of sintilimab included pneumonia, diarrhea, colitis, hepatitis, nephritis, endocrinology diseases, skin AEs, infusion reactions, and other immune-related AEs (27). The AEs were defined according to the Common Terminology Criteria for Adverse Events (CTCAEs) from the National Cancer Institute and were coded using the Medical Dictionary for Regulatory Activities (69, 70). The CTCAE have five grades, referring to mild, moderate, severe, life-threatening AEs or death, in an ascending order.

As listed in **Table 1**, treatment-related AEs (TRAEs) were reported in most of the patients in major clinical studies and the incidences range from 52.5 to 100% among them. In ORIENT-1, TRAEs occurred in 92.7% (89/96) patients, and grade 3–5 TRAEs occurred in 17.7% (17/96) patients, without TARE-related death (26). In CHECKMATE-205, TRAEs occurred in 98.8% (79/80) patients, grade 3–5 TRAEs occurred in 41.3% (32/80) patients, and there was one TARE-related death (29). In KEYNOTE-087, no patient died of TRAEs although the occurrence rate of TRAEs and grade 3–5 TRAEs were not available (30). Thus, sintilimab caused less toxicities in patients with r/r cHL than nivolumab and was proved safe. In ChiCTR-OIC-17013726, the incidence of all grade TRAEs was 52.5% (21/40); the incidence of 3–5 TRAEs was 10.0% (4/40), and the rate of TRAE-related death was 2.5% (1/40) (36). In NCT02259621, the incidences and rate were 22.7% (5/22), 4.5% (1/22), and 0 (39). In MK3475-223, the data were not available (40, 41). Although the safety profile of sintilimab in NSCLC seems worse than nivolumab when used preoperatively, the conclusion needs more evidence since the sample size of the latter is small.

The safety of ICIs has been evaluated in a previous study revealing an incidence rate varying between 54 and 76% for all AEs (71). When sintilimab was used alone, TRAEs occurred in more than 58.0% (120/207) patients, with 13.0% (27/207) grade 3 or higher TRAEs and one death (0.5%, 1/207) (25, 26, 36, 64, 72). For nivolumab, a pooled analysis of four major clinical trials on NSCLC showed that 69.4% (461/664) of patients had TRAEs and that grade 3–5 treatment-related adverse events occurred in 13.9% (92/664) of patients; overall, there are 0.9% (6/664) deaths (73). For pembrolizumab in NSCLC, the rate of TRAEs was 66.3% (1304/1967), with 15.2% (299/1967) grade 3 or higher TRAEs when used alone, and at least 1.0% (20/1967) deaths (74–77). Sintilimab seems safer than nivolumab or pembrolizumab when used alone because of its lower TRAE incidence and death rate. However, the conclusion may not be accurate because the studies of sintilimab involve various cancers in addition to NSCLC.

When sintilimab was combined with chemotherapy (pemetrexed and platinum), TRAEs occurred in 99.6% (265/266) of patients, with 61.7% (164/266) grade 3 or higher TRAEs and 2.3% (6/266) of AE-related deaths compared to those of 100% (131/131), 58.8% (77/131), and 6.9% (9/131) in the control group (43). When pembrolizumab was combined with pemetrexed and platinum, the rate of TRAEs reached 99.8% (404/405), with 71.9% (291/405) grade 3 or higher TRAEs, and 29 deaths (7.2%, 29/405) (44). Preliminarily, when combined with pemetrexed and platinum, the incidences of TRAEs are similar but the incidence of ≥3 TRAEs and the death rate are lower for sintilimab. Thus, sintilimab had better safety profiles compared to nivolumab or pembrolizumab when used alone in r/r cHL and when combined with pemetrexed and platinum in advanced NSCLC.

Besides, there were also AEs not mentioned in clinical studies or in the drug instructions. In the study by Xing et al., a 66-year-old lung adenocarcinoma patient received two doses of sintilimab as monotherapy and developed myositis–myasthenia gravis overlap syndrome complicated with myasthenia crisis; the overlap syndrome was cured after supportive therapies such as plasma exchange, mechanical ventilation, and immunosuppressive therapy (78). In an HCC patient, sintilimab administration resulted in autoimmune diabetes at 24 weeks after injection (79). In other reported cases, patients developed cytokine release syndrome, pulmonary fibrosis, hypothyroid myopathy, encephalitis during or after sintilimab administration (80–83). All syndromes in these patients were relieved after drug withdrawal and glucocorticoid treatment; there were no deaths in these cases.

## Discussion

**Figure 1:**
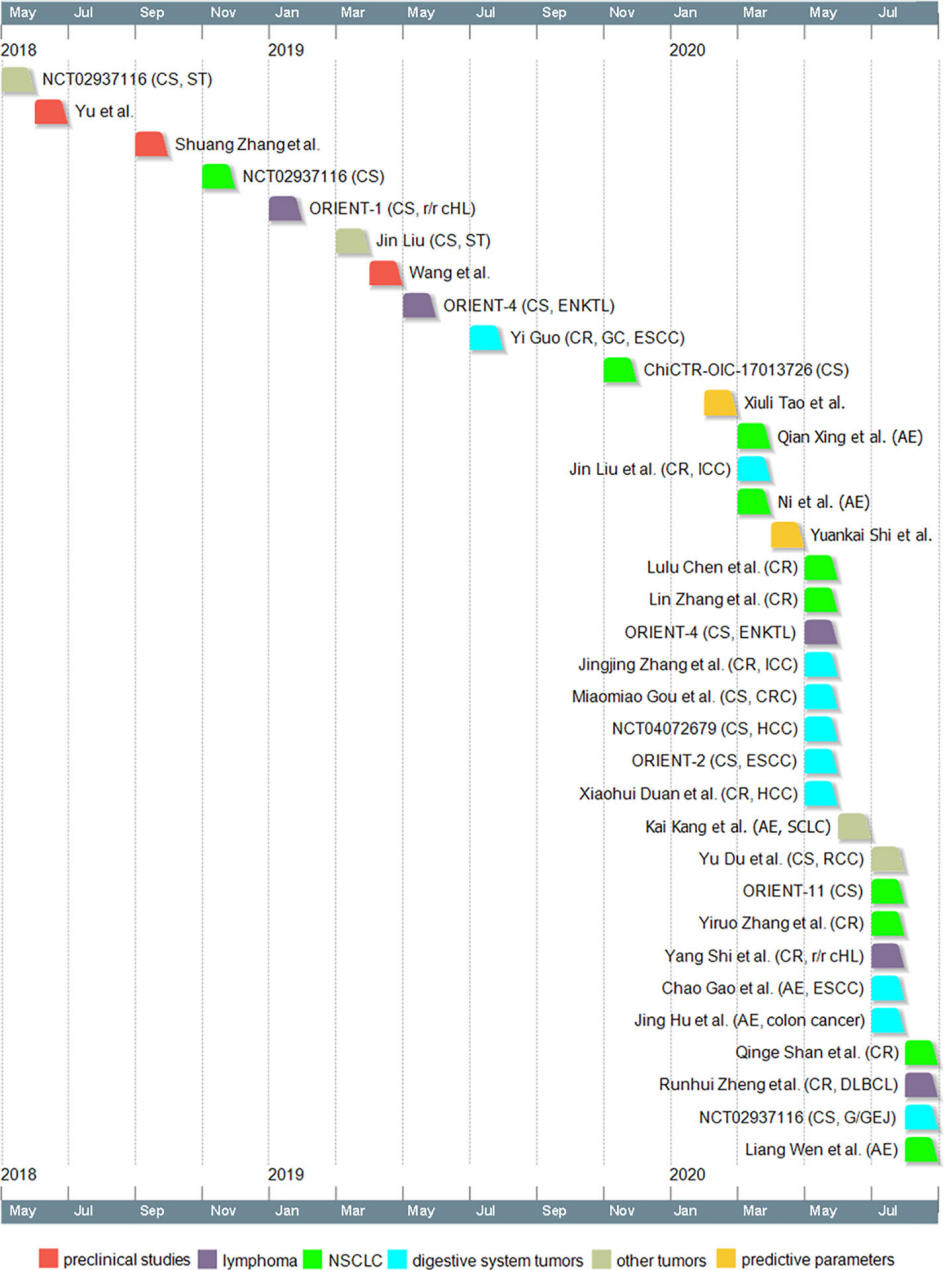
Timeline of sintilimab-related studies. Dates indicate the time when the study was first available online. CS, clinical study; ST, solid tumors; r/r cHL, relapsed or refractory classical Hodgkin lymphoma; ENKTL, extranodal NK/T cell lymphoma; CR, case report; GC, gastric cancer; ESCC, esophageal squamous cell carcinoma; AE, case reports on adverse effects; ICC, intrahepatic cholangiocarcinoma; CRC, colorectal cancer; HCC, hepatocellular carcinoma; SCLC, small cell lung cancer; RCC, renal cell carcinoma; DLBCL, diffuse large B-cell lymphoma; G/GEJ, gastric/gastroesophageal junction adenocarcinoma; NSCLC, non-small cell lung cancer.

Sintilimab is a domestic PD-1 antibody in China and has received its approval on 24 December 2018 for the treatment of r/r cHL. The drug has shown promising anti-tumor effect on various cancer types in a variety of studies. The studies related to sintilimab were summarized in a timeline in **Figure 1**. The results of these studies have indicated a bright future for sintilimab.

However, there are obvious limitations. First of all, there is lack of direct head-to-head comparisons to other PD-1 inhibitors like nivolumab and pembrolizumab. Secondly, among the studies included, complete data of some studies, such as ORIENT-2 and ORIENT-4, are not available. More importantly, almost all of the patients included are native Chinese, and there is only one registered study outside China (NCT03748134/ORIENT-15) (66). The efficacy of this drug remains to be explored in people of other races. Future studies addressing these questions could be implemented.

Despite the limitations, there are some aspects worth expecting. First, there are a number of ongoing clinical studies assessing sintilimab for treating various cancers (66). In addition, in a health technology assessment, sintilimab got a total score of 68, compared to those of 75 for nivolumab, 62 for pembrolizumab, 66 for toripalimab, and 58 for camrelizumab (84). Furthermore, sintilimab has a lower cost (approximately 24,000 USD/year) than other well-studied PD-1 inhibitors (approximately 87,000 USD/year for pembrolizumab and 63,000 USD/year for nivolumab). Considering the different economic background of Chinese patients from that of patients in developed western countries, the lower cost of sintilimab can sometimes be decisive in their choices of cancer treatment.

## Conclusion

In conclusion, sintilimab has been preliminarily proved effective and safe in the treatment of various cancers. Although the efficacy of sintilimab in these cancers still needs to be validated in further research, it is hopeful that sintilimab will be approved with more indications in the near future. Our review has provided clinical physicians with a potential choice for the treatment of cancers in the future.

## Author Contributions

LZ: conceptualization. LZ and WM: writing, visulization, and original draft. WJ and QG: review and editing. QG: funding. All authors contributed to the article and approved the submitted version.

## Funding

This work was supported by National Natural Science Foundation of China granted to QG (81770095).

## Conflict of Interest

The authors declare that the research was conducted in the absence of any commercial or financial relationships that could be construed as a potential conflict of interest.
